# Methyl (*E*)-3-(2-formyl­phen­oxy)acrylate

**DOI:** 10.1107/S1600536814010617

**Published:** 2014-05-24

**Authors:** S. Karthikeyan, K. Sethusankar, R. Selvakumar, M. Bakthadoss

**Affiliations:** a Department of Physics, RKM Vivekananda College (Autonomous), Chennai 600 004, India; b Department of Organic Chemistry, University of Madras, Maraimalai Campus, Chennai 600 025, India

## Abstract

In the title compound, C_11_H_10_O_4_, the methyl acrylate sub­stituent adopts an extended *E* conformation with all torsion angles close to 180°. The conformation of the keto group with respect to the olefinic double bond is typically *S-trans*. In the crystal, mol­ecules are linked *via* pairs of C—H⋯O hydrogen bonds, forming inversion dimers with an *R*_2_^2^(8) graph-set motif. The dimers are further linked *via* C—H⋯O hydrogen bonds, forming chains along [001], which enclose *R*_3_^2^(16) graph-set ring motifs. The keto group O atomaccepts two C—H⋯O interactions.

## Related literature   

For applications of acrylate derivatives, see: Xiao *et al.* (2008 ▶); De *et al.* (2011 ▶); Sharma (2011 ▶). For related crystal structures, see: Karthikeyan *et al.* (2012 ▶). For *E*-conformation aspects, see: Dunitz & Schweizer (1982 ▶). For resonance effects of acrylate, see: Merlino (1971 ▶); Varghese *et al.* (1986 ▶). For graph-set motif notation, see: Bernstein *et al.* (1995 ▶).
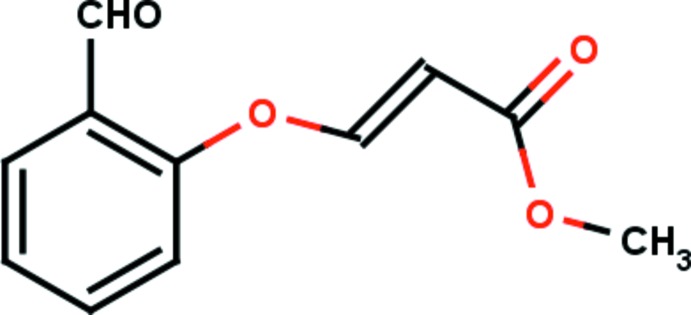


## Experimental   

### 

#### Crystal data  

C_11_H_10_O_4_
*M* *_r_* = 206.19
Monoclinic, 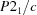

*a* = 17.7458 (8) Å
*b* = 4.0629 (2) Å
*c* = 14.5745 (7) Å
β = 107.868 (3)°
*V* = 1000.13 (8) Å^3^
*Z* = 4
Mo *K*α radiation
μ = 0.11 mm^−1^
*T* = 293 K
0.20 × 0.15 × 0.10 mm

#### Data collection  

Bruker SMART APEXII CCD diffractometer
13052 measured reflections
2015 independent reflections
1523 reflections with *I* > 2σ(*I*)
*R*_int_ = 0.027

#### Refinement  

*R*[*F* ^2^ > 2σ(*F* ^2^)] = 0.045
*wR*(*F* ^2^) = 0.155
*S* = 1.06
2015 reflections
137 parameters
H-atom parameters constrained
Δρ_max_ = 0.23 e Å^−3^
Δρ_min_ = −0.17 e Å^−3^

### 

Data collection: *APEX2* (Bruker, 2008 ▶); cell refinement: *SAINT* (Bruker, 2008 ▶); data reduction: *SAINT*; program(s) used to solve structure: *SHELXS97* (Sheldrick, 2008 ▶); program(s) used to refine structure: *SHELXL97* (Sheldrick, 2008 ▶); molecular graphics: *ORTEP-3 for Windows* (Farrugia, 2012 ▶) and *Mercury* (Macrae *et al., *2008 ▶); software used to prepare material for publication: *SHELXL97* and *PLATON* (Spek, 2009 ▶).

## Supplementary Material

Crystal structure: contains datablock(s) global, I. DOI: 10.1107/S1600536814010617/su2732sup1.cif

Structure factors: contains datablock(s) I. DOI: 10.1107/S1600536814010617/su2732Isup2.hkl

Supporting information file. DOI: 10.1107/S1600536814010617/su2732Isup3.cml

CCDC reference: 1001914

Additional supporting information:  crystallographic information; 3D view; checkCIF report

## Figures and Tables

**Table 1:** Hydrogen-bond geometry (Å, °)

*D*—H⋯*A*	*D*—H	H⋯*A*	*D*⋯*A*	*D*—H⋯*A*
C9—H9⋯O2^i^	0.93	2.54	3.440 (2)	164
C8—H8⋯O4^ii^	0.93	2.61	3.529 (2)	171
C11—H11*C*⋯O2^iii^	0.96	2.63	3.578 (2)	168

Symmetry codes: (i) 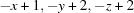; (ii) 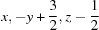; (iii) 
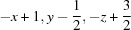
.

**Fig. 1.**
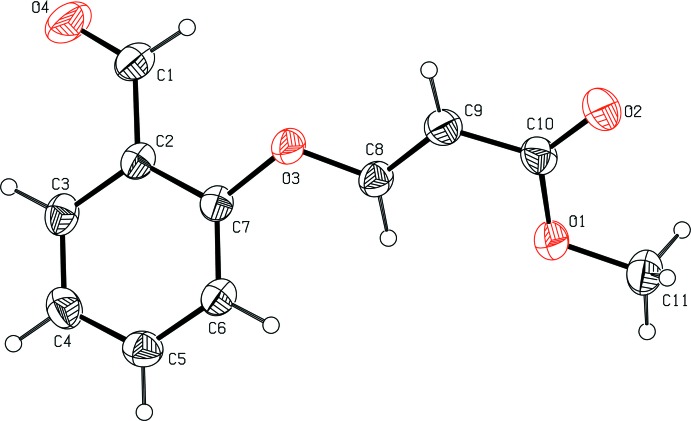
The molecular structure of the title molecule, with atom labelling. Displacement ellipsoids are drawn at the 30% probability level.

**Fig. 2.**
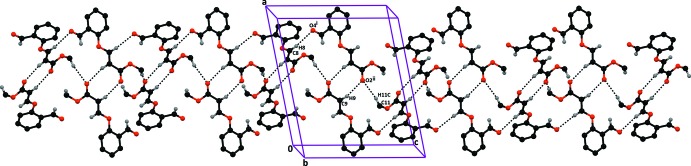
The crystal packing of the title compound viewed along the b axis, showing the formation of the R22(8) graph-set motif. The dimers are further consolidated by R23(16) graph-set ring motifs. Hydrogen bonds are shown as dashed lines (see Table 1 for details; H atoms not involved in these interactions have been omitted for clarity).)

## supplementary crystallographic information

### 1. Comment

Cinnamic acid derivatives have received attention in medicinal research as traditional as well as recent synthetic antitumor agents (De *et al.*, 2011). They also posses significant antibacterial activities against *staphylococcus aureus* (Xiao *et al.*, 2008). Different substitutions on the basic moiety lead to various pharmacological activities, such as antioxidant, hepatoprotective, anxiolytic, insect repellent, antidiabetic, and anticholesterolemic (Sharma, 2011).

In the title molecule, Fig. 1, the methyl acrylate group is essentially planar, with a maximum deviation of 0.0264 (19) Å for atom C9. Its mean plane forms a dihedral angle of 31.74 (6)° with the benzene ring (C2—C7). The molecular dimensions are in excellent agreement with the those reported for a closely related compound (Karthikeyan *et al.*, 2012).

The configuration of the keto group with respect to the olefinic double bond is typically S-*trans*, with the O2═C10—C9═C8 torsion angle = 178.78 (19)°. The methyl acrylate group adopts an extended *E* conformation with torsion angles C8═C9—C10═O2 = 178.78 (19)°, C8═ C9—C10—O1 = -1.2 (3)°, C9—C10—O1—C11 = -178.82 (16)° and O2═ C10—O1—C11 = 1.2 (3)°. The extended conformation is supported by the fact that the bond angles involving carbonyl O atoms are invariably enlarged (Dunitz & Schweizer, 1982).

The significant difference in the bond lengths C10—O1 = 1.342 (2) Å and C11—O1 = 1.438 (2) Å is attributed to a partial contribution from the O^-^—C═O^+^—C resonance structure of the O2═C10—O1—C11 group (Merlino, 1971). This feature, commonly observed for the carboxylic ester group of substituents in various compounds gives average values of 1.340 Å and 1.447 Å, respectively (Varghese *et al.*, 1986).

The crystal packing (Fig. 2 and Table 1) is stabilized by C—H···O intermolecular interactions. The molecules are linked into inversion dimers *via* C9—H9···O2 interactions resulting in an *R*^2^_2_(8) graph-set motif (Bernstein *et al.*, 1995). The dimers are further consolidated by *R*^2^_3_(16) graph-set ring motifs *via* C8—H8···O4 and C11—H11C···O2 interactions resulting in chains of molecules running parallel to the c axis; the keto group O atom (O2) is involved in bifurcated hydrogen bonding.

### 2. Experimental

Salicylaldehyde (1 mmol) was dissolved in an aqueous solution of K_2_CO_3_ (1 mmol) and methyl propiolate (1 mmol) was added. The reaction mixture was stirred vigorously at room temperature. A turbid solution was formed by consumption of salicylaldehyde (monitored by TLC) in 5 min, the reaction mixture then became clear. The title compound was precipitated as a solid in water. The product was isolated by filtration without further purification [Yield 75%]. Block-like colourless crystals were obtained by slow evaporation of a solution in ethylacetate.

### 3. Refinement

The H atoms could all be located in difference electron-density maps. In the final cycles of refinement they were treated as riding atoms: C—H = 0.93 and 0.96 Å for CH and CH_3_ H atoms, respectively, with *U*_iso_(H) = 1.5 *U*_eq_(C– methyl) and = 1.2*U*_eq_(C) for other H atoms.

### Crystal data

**Table:**

C_11_H_10_O_4_	*F*(000) = 432
*M**_r_* = 206.19	*D*_x_ = 1.369 Mg m^−^^3^
Monoclinic, *P*2_1_/*c*	Mo *K*α radiation, λ = 0.71073 Å
Hall symbol: -p 2ybc	Cell parameters from 2015 reflections
*a* = 17.7458 (8) Å	θ = 1.2–26.3°
*b* = 4.0629 (2) Å	µ = 0.11 mm^−^^1^
*c* = 14.5745 (7) Å	*T* = 293 K
β = 107.868 (3)°	Block, colorless
*V* = 1000.13 (8) Å^3^	0.20 × 0.15 × 0.10 mm
*Z* = 4	

### Data collection

**Table:**

Bruker SMART APEXII CCD diffractometer	1523 reflections with *I* > 2σ(*I*)
Radiation source: fine-focus sealed tube	*R*_int_ = 0.027
Graphite monochromator	θ_max_ = 26.3°, θ_min_ = 1.2°
ω scans	*h* = −22→21
13052 measured reflections	*k* = −5→5
2015 independent reflections	*l* = −18→18

### Refinement

**Table:**

Refinement on *F*^2^	Primary atom site location: structure-invariant direct methods
Least-squares matrix: full	Secondary atom site location: difference Fourier map
*R*[*F*^2^ > 2σ(*F*^2^)] = 0.045	Hydrogen site location: inferred from neighbouring sites
*wR*(*F*^2^) = 0.155	H-atom parameters constrained
*S* = 1.06	*w* = 1/[σ^2^(*F*_o_^2^) + (0.1001*P*)^2^ + 0.0957*P*] where *P* = (*F*_o_^2^ + 2*F*_c_^2^)/3
2015 reflections	(Δ/σ)_max_ < 0.001
137 parameters	Δρ_max_ = 0.23 e Å^−^^3^
0 restraints	Δρ_min_ = −0.17 e Å^−^^3^

### Special details

**Table:**

Geometry. All e.s.d.'s (except the e.s.d. in the dihedral angle between two l.s. planes) are estimated using the full covariance matrix. The cell e.s.d.'s are taken into account individually in the estimation of e.s.d.'s in distances, angles and torsion angles; correlations between e.s.d.'s in cell parameters are only used when they are defined by crystal symmetry. An approximate (isotropic) treatment of cell e.s.d.'s is used for estimating e.s.d.'s involving l.s. planes.
Refinement. Refinement of *F*^2^ against ALL reflections. The weighted *R*-factor *wR* and goodness of fit *S* are based on *F*^2^, conventional *R*-factors *R* are based on *F*, with *F* set to zero for negative *F*^2^. The threshold expression of *F*^2^ > σ(*F*^2^) is used only for calculating *R*-factors(gt) *etc*. and is not relevant to the choice of reflections for refinement. *R*-factors based on *F*^2^ are statistically about twice as large as those based on *F*, and *R*- factors based on ALL data will be even larger.

### Fractional atomic coordinates and isotropic or equivalent isotropic displacement parameters (Å^2^)

**Table:**

	*x*	*y*	*z*	*U*_iso_*/*U*_eq_	
C1	0.19462 (11)	0.6483 (5)	1.09554 (11)	0.0570 (5)	
H1	0.2426	0.7588	1.1079	0.068*	
C2	0.16126 (8)	0.4928 (4)	1.00055 (10)	0.0437 (4)	
C3	0.08802 (9)	0.3378 (4)	0.97865 (12)	0.0518 (5)	
H3	0.0621	0.3250	1.0252	0.062*	
C4	0.05341 (10)	0.2034 (5)	0.88901 (13)	0.0574 (5)	
H4	0.0040	0.1033	0.8746	0.069*	
C5	0.09250 (10)	0.2182 (5)	0.82056 (12)	0.0545 (5)	
H5	0.0692	0.1265	0.7600	0.065*	
C6	0.16554 (9)	0.3671 (4)	0.84083 (11)	0.0484 (4)	
H6	0.1918	0.3734	0.7946	0.058*	
C7	0.19943 (8)	0.5068 (4)	0.93033 (10)	0.0425 (4)	
C8	0.30241 (9)	0.7868 (4)	0.88985 (11)	0.0463 (4)	
H8	0.2706	0.8014	0.8260	0.056*	
C9	0.37579 (9)	0.8937 (5)	0.91462 (12)	0.0542 (5)	
H9	0.4075	0.8649	0.9780	0.065*	
C10	0.40974 (9)	1.0549 (5)	0.84737 (12)	0.0516 (4)	
C11	0.39059 (12)	1.2299 (6)	0.68764 (14)	0.0655 (5)	
H11A	0.4103	1.4452	0.7097	0.098*	
H11B	0.3494	1.2484	0.6272	0.098*	
H11C	0.4329	1.0976	0.6794	0.098*	
O1	0.35946 (7)	1.0781 (3)	0.75754 (8)	0.0598 (4)	
O2	0.47632 (7)	1.1596 (4)	0.86851 (10)	0.0723 (5)	
O3	0.27287 (6)	0.6544 (3)	0.95771 (7)	0.0541 (4)	
O4	0.16367 (9)	0.6413 (5)	1.15808 (9)	0.0821 (5)	

### Atomic displacement parameters (Å^2^)

**Table:**

	*U*^11^	*U*^22^	*U*^33^	*U*^12^	*U*^13^	*U*^23^
C1	0.0536 (10)	0.0738 (13)	0.0448 (8)	0.0075 (8)	0.0171 (7)	0.0039 (8)
C2	0.0403 (8)	0.0498 (10)	0.0419 (8)	0.0095 (6)	0.0142 (6)	0.0084 (7)
C3	0.0432 (9)	0.0604 (11)	0.0562 (9)	0.0071 (7)	0.0220 (7)	0.0119 (8)
C4	0.0442 (9)	0.0590 (11)	0.0665 (11)	−0.0046 (8)	0.0134 (8)	0.0077 (8)
C5	0.0531 (10)	0.0542 (10)	0.0504 (9)	0.0009 (8)	0.0074 (7)	0.0007 (8)
C6	0.0485 (9)	0.0547 (10)	0.0443 (8)	0.0048 (7)	0.0175 (7)	0.0049 (7)
C7	0.0357 (7)	0.0485 (10)	0.0429 (7)	0.0054 (6)	0.0115 (6)	0.0082 (6)
C8	0.0411 (8)	0.0565 (10)	0.0435 (8)	0.0025 (7)	0.0162 (6)	0.0013 (7)
C9	0.0425 (8)	0.0710 (12)	0.0490 (9)	−0.0019 (8)	0.0137 (7)	−0.0027 (8)
C10	0.0383 (8)	0.0609 (11)	0.0580 (9)	−0.0018 (7)	0.0184 (7)	−0.0063 (8)
C11	0.0639 (11)	0.0721 (13)	0.0669 (11)	−0.0039 (10)	0.0297 (9)	0.0095 (10)
O1	0.0487 (7)	0.0767 (9)	0.0553 (7)	−0.0123 (6)	0.0181 (5)	0.0033 (6)
O2	0.0431 (7)	0.1021 (13)	0.0731 (8)	−0.0180 (7)	0.0197 (6)	−0.0050 (7)
O3	0.0403 (6)	0.0800 (9)	0.0427 (6)	−0.0071 (5)	0.0139 (5)	0.0042 (5)
O4	0.0787 (10)	0.1250 (15)	0.0509 (7)	−0.0008 (9)	0.0322 (7)	−0.0084 (7)

### Geometric parameters (Å, º)

**Table:**

C1—O4	1.200 (2)	C7—O3	1.3778 (18)
C1—C2	1.471 (2)	C8—C9	1.314 (2)
C1—H1	0.9300	C8—O3	1.3640 (18)
C2—C3	1.390 (2)	C8—H8	0.9300
C2—C7	1.3910 (19)	C9—C10	1.454 (2)
C3—C4	1.375 (3)	C9—H9	0.9300
C3—H3	0.9300	C10—O2	1.2035 (19)
C4—C5	1.380 (2)	C10—O1	1.342 (2)
C4—H4	0.9300	C11—O1	1.438 (2)
C5—C6	1.378 (2)	C11—H11A	0.9600
C5—H5	0.9300	C11—H11B	0.9600
C6—C7	1.380 (2)	C11—H11C	0.9600
C6—H6	0.9300		
			
O4—C1—C2	123.98 (17)	O3—C7—C2	115.83 (13)
O4—C1—H1	118.0	C6—C7—C2	120.61 (14)
C2—C1—H1	118.0	C9—C8—O3	120.00 (14)
C3—C2—C7	118.80 (14)	C9—C8—H8	120.0
C3—C2—C1	119.24 (14)	O3—C8—H8	120.0
C7—C2—C1	121.91 (15)	C8—C9—C10	122.98 (15)
C4—C3—C2	120.77 (15)	C8—C9—H9	118.5
C4—C3—H3	119.6	C10—C9—H9	118.5
C2—C3—H3	119.6	O2—C10—O1	122.10 (15)
C3—C4—C5	119.52 (16)	O2—C10—C9	124.32 (16)
C3—C4—H4	120.2	O1—C10—C9	113.58 (14)
C5—C4—H4	120.2	O1—C11—H11A	109.5
C6—C5—C4	120.83 (15)	O1—C11—H11B	109.5
C6—C5—H5	119.6	H11A—C11—H11B	109.5
C4—C5—H5	119.6	O1—C11—H11C	109.5
C5—C6—C7	119.46 (15)	H11A—C11—H11C	109.5
C5—C6—H6	120.3	H11B—C11—H11C	109.5
C7—C6—H6	120.3	C10—O1—C11	115.85 (13)
O3—C7—C6	123.51 (13)	C8—O3—C7	120.10 (12)
			
O4—C1—C2—C3	2.7 (3)	C3—C2—C7—C6	−0.5 (2)
O4—C1—C2—C7	−179.85 (17)	C1—C2—C7—C6	−178.03 (15)
C7—C2—C3—C4	−0.6 (2)	O3—C8—C9—C10	−176.28 (16)
C1—C2—C3—C4	176.94 (17)	C8—C9—C10—O2	178.78 (19)
C2—C3—C4—C5	1.0 (3)	C8—C9—C10—O1	−1.2 (3)
C3—C4—C5—C6	−0.3 (3)	O2—C10—O1—C11	1.2 (3)
C4—C5—C6—C7	−0.9 (3)	C9—C10—O1—C11	−178.82 (16)
C5—C6—C7—O3	178.55 (15)	C9—C8—O3—C7	−172.25 (16)
C5—C6—C7—C2	1.3 (2)	C6—C7—O3—C8	26.2 (2)
C3—C2—C7—O3	−178.02 (14)	C2—C7—O3—C8	−156.37 (14)
C1—C2—C7—O3	4.5 (2)		

### Hydrogen-bond geometry (Å, º)

**Table:**

*D*—H···*A*	*D*—H	H···*A*	*D*···*A*	*D*—H···*A*
C9—H9···O2^i^	0.93	2.54	3.440 (2)	164
C8—H8···O4^ii^	0.93	2.61	3.529 (2)	171
C11—H11*C*···O2^iii^	0.96	2.63	3.578 (2)	168

Symmetry codes: (i) −*x*+1, −*y*+2, −*z*+2; (ii) *x*, −*y*+3/2, *z*−1/2; (iii) −*x*+1, *y*−1/2, −*z*+3/2.
